# Determinants of Treatment Adherence and Health Outcomes in Patients With Type 2 Diabetes and Hypertension in a Low-Income Urban Agglomerate in Delhi, India: A Qualitative Study

**DOI:** 10.7759/cureus.34826

**Published:** 2023-02-09

**Authors:** Nandini Sharma, Warisha Mariam, Saurav Basu, Rahul Shrivastava, Shivani Rao, Pragya Sharma, Sandeep Garg

**Affiliations:** 1 Department of Community Medicine, Maulana Azad Medical College, New Delhi, IND; 2 Department of Medicine, Indian Institute of Public Health - Delhi, Public Health Foundation of India, New Delhi, IND; 3 Department of Public Health, Dehradun Institute of Technology (DIT) University, Dehradun, IND; 4 Department of Biotechnology, National Biopharma Mission, Biotechnology Industry Research Assistance Council (BIRAC), New Delhi, IND; 5 Community Medicine, Maulana Azad Medical College, New Delhi, IND; 6 Department of Internal Medicine, Maulana Azad Medical College, New Delhi, IND

**Keywords:** noncommunicable disease, treatment pathways, qualitative study, diabetes, adherence

## Abstract

Background

Diabetes and hypertension (HTN) are increasing threats to global public health. Despite evidence of effective management of diabetes and HTN by medications that help in the prevention and reducing mortality of the disease, a large proportion of people either remain undiagnosed or untreated, especially in low-resource countries. This study was conducted to explore the patient treatment pathway and their health-seeking behavior in a low-income urban area.

Methodology

We conducted 45 in-depth interviews of adult patients affected by type 2 diabetes mellitus (DM) and/or HTN on treatment for at least two years and attended the weekly clinic catering to an urban resettlement colony in the Northeast district of Delhi. Interviews were conducted and transcribed into Hindi and translated into English. Data analysis was done using Microsoft Excel. The patient treatment pathways were mapped, and their health-seeking behavior, treatment adherence, and experiences were described.

Results

Most patients reported taking treatment from the government primary health facilities due to optimal healthcare accessibility as the prescribed drugs for DM/HTN control were available free of cost at these healthcare facilities. Those who visited private facilities thought of shorter waiting times and the quality of drugs. Patients also had little knowledge of complications of diabetes and hypertensive disorders. Nearly 25% of patients had poor adherence to the medications, and lifestyle modification was rarely practiced by patients although they were aware of the same.

Conclusions

Expanding the role of community health workers or volunteers in providing information on noncommunicable diseases might help improve patient treatment pathways to care.

## Introduction

Noncommunicable diseases (NCDs) after an ongoing epidemiological and demographic transition globally account for an estimated two-thirds of the global disease burden in 2019 compared to one-third in 1990 [1]. NCDs being incurable, the failure to achieve optimal control due to nonadherence to prescribed treatment and lifestyle modifications accelerates the premature incidence of complications, reduced quality of life, and excess mortality, which is disproportionately concentrated in the low- and middle-income countries (LMICs) [2].

Appropriate care-seeking behaviors in the context of chronic diseases reflect adherence to self-care practices and a continuum of care with licensed healthcare providers to enable successful disease management and achieve improved health outcomes [3]. However, both restricted healthcare system accessibility and adverse social determinants of health such as low socioeconomic status (SES), female gender, illiteracy, and low education contribute to inappropriate care-seeking, which correlates with poor health outcomes [4,5]. Elderly populations have a significantly higher prevalence of NCDs but are also at increased risk of deficient care-seeking, particularly in the absence of familial and social support [6,7].

Public primary healthcare (PHC) services in developing countries are considered the cornerstone in the provision of free and equitable delivery of healthcare services for preventive, promotive, and curative health [8]. PHC services through qualified general-duty medical officers are adequate for most patients with DM and HTN in achieving their recommended health targets and specialist consultation is required in only a fraction of the cases [9,10]. Urban healthcare services have, however, disproportionately focused on secondary and tertiary-based curative services, which can deprive the most vulnerable urban poor populations living in urban slums and resettlement colonies, of maintaining continuum of care for chronic disease management [11,12].

India has globally the second-highest diabetes burden in the world accounting for over 69 million patients [13]. Hypertension (HTN) is estimated to directly account for 57% of all stroke deaths and 24% of all coronary heart disease (CHD) deaths in India [14]. PHC services in urban areas of the country at urban primary health centers (UPHCs) that are staffed mostly by allopathy medical officers who at least have an MBBS degree with or without an additional specialist (MD/MS) degree. All services are provided free of cost without any user charges inclusive of consultation, generic drugs from an essential drug list, and select laboratory investigations. However, a referral from UPHCs is not mandatory for availing specialist outpatient consultation services from higher facilities consisting of secondary and tertiary care government hospitals. A major reason for high out-of-pocket expenses incurred in Indian outpatient health settings among patients with NCDs is the lack of assured and sustained availability of diagnostics and drugs and the absence of public health insurance coverage in outpatient health departments [15,16].

There is a paucity of information on the patterns of health and treatment-seeking behavior of patients with DM and HTN in low-income urban agglomerates of Delhi. Thus, this study was done to identify treatment-seeking behavior pathways of patients with diabetes and HTN in a low-income urban area of Delhi, India.

## Materials and methods

Study design and setting

This was a qualitative study conducted in the outpatient settings of a Delhi Government Dispensary (DGD), also the primary health facility catering to an urban resettlement and slum population in the Northeast district of Delhi. The site was purposively selected as it is the field practice of the Department of Community Medicine of a major government hospital in Delhi.

Adult patients with type 2 diabetes mellitus (DM) and/or HTN on treatment for at least two years and attending the weekly NCD clinic at the DGD were eligible to participate in this study. Patients with preexisting serious comorbidities such as cancer, advanced renal failure requiring dialysis, advanced COPD, and advanced cardiovascular disease were excluded.

Data collection

Data were collected through in-depth interviews, with the participants each lasting nearly 20-25 minutes. A semistructured interview guide adapted from a previous study in low-resource settings was used to assess patients' perspectives on treatment adherence and diabetes and HTN-related health outcomes [17,18]. A maximum of five participants were interviewed in a single day from August 2021 to November 2021. A short, structured patient interview schedule was used to collect sociodemographic and clinical information from the participants. The SES was measured with the modified Kuppuswamy scale updated for the 2021 consumer price index [19].

The study involved in-depth interviews conducted by trained field investigators (one male and one female). Interviews were conducted in the local language, Hindi, and audio-recorded following written informed consent. All interviews were transcribed in Hindi by the interviewers themselves, translated into English, reviewed line by line, and double-checked by two investigators.

Ethical considerations

The study was approved by the Institutional EethicsCcommittee, Maulana Azad Medical College, and Associated Hospitals, New Delhi (F.1/IEC/MAMC/(84/02/2021/No383). All the study participants provided written and informed consent. The study team made efforts to ensure interviews were undertaken while maintaining the privacy and confidentiality of the participants.

Data analysis

To ensure the validity and reliability of analyses, the interview transcripts were separately and independently coded by two of the investigators and any differences were resolved by discussion. Manual coding was done using an inductive approach using Microsoft Excel including line-by-line analysis. Thematic analysis was used for the identification of recurrent themes by applying the standardized Erlingsson and Brysiewicz framework [20].

Participants' recruitment ceased following thematic saturation. Interviews were recorded and transcribed in full. Each transcript contains the assigned participant number and age, sex, health condition, and comorbidity status of the participant.

## Results

The study participants included 21 diabetes and HTN comorbidity patients, nine diabetes patients without HTN, and 15 hypertensive patients without diabetes. The mean (±SD) age of the study participants was 54.7 (±10.9) years. There were 32 women and 13 men participants (Table 1).

**Table 1:** Sociodemographic characteristics of study participants of an urban resettlement colony of Delhi, 2021 (N = 45). IQR, interquartile range

Characteristics	Number	Percentage
Age		
Mean (SD)	54.9 (10.7)	
Median (IQR)	56 (45-63)	
30-39 years	5	11.1
40-49 years	7	15.6
50-59 years	13	28.9
>= 60 years	20	44.4
Gender		
Female	32	71
Male	13	29
Education status		
Illiterate	28	62
Literate	17	38
Occupation		
Homemaker	28	62.2
Unemployed	2	4.4
Semiskilled worker	6	13.3
Unskilled worker	9	20.0
Socioeconomic status		
Lower	11	24.4
Upper lower	23	51.1
Lower middle	9	20.0
Upper middle	2	4.4
Upper	0	0

Nearly three in four participants belonged to the lower SES and a majority were illiterate and none had any health insurance meeting outpatient expenses. None of the patients with DM were currently on insulin therapy.

A majority (53.6%) of patients with DM had poor glycemic control (*n *= 30). Routine follow-up investigations for monitoring the health of patients with DM/HTN were delayed in most of the participants (Table 2).

**Table 2:** Behavioral characteristics toward HTN and diabetes management among study participants of an urban resettlement colony of Delhi, 2021 (N = 45). *Not mutually exclusive. SMBG, self-monitoring of blood glucose; HTN, hypertension; HbA1c, hemoglobin A1c; KFT, kidney function test

Characteristics	Number	Percentage	
Glycemic control (*n* = 30)			
Good	14	46.7	
Poor	16	53.3	
BP control (*n *= 36)			
Good	21	58.3	
Poor	15	41.7	
Adherence to investigation			
Plasma glucose			
Yes (within 90 days)	20	44.4	
Delayed	11	22.4	
Not done within one year	7	15.6	
Never done	7	15.6	
SMBG			
Yes (within seven days)	4	8.9	
Delayed	3	6.7	
Not done within one year	2	4.4	
Never done	36	80.0	
HbA1c			
Yes (within 180 days)	2	4.4	
Delayed	7	15.6	
Not done within one year	8	17.8	
Never done	28	62.2	
Lipid profile			
Yes (within one year)	3	6.7	
Delayed	6	13.3	
Not done within one year	14	31.1	
Never done	22	48.9	
KFT			
Yes (within one year)	4	8.9	
Delayed	4	8.9	
Not done within one year	17	37.8	
Never done	20	44.4	
Adherence to medications			
Do you ever forget to take your medicine?			
Yes	16	35.6	
No	29	64.4	
Are you careless at times about taking your medicine?			
Yes	11	24.4	
No	34	75.6	
When you feel better, do you sometimes stop taking your medicine?			
Yes	9	20.0	
No	36	80.0	
Sometimes you feel worse, when you take the medicine, do you stop taking it?			
Yes	3	6.7	
No	42	93.3	
Health-seeking behavior for medication*			
Government Health Facility	41	91.1	
Private Health Facility	13	28.9	
Jan Aushadhi Store	2	4.4	

**Figure 1:**
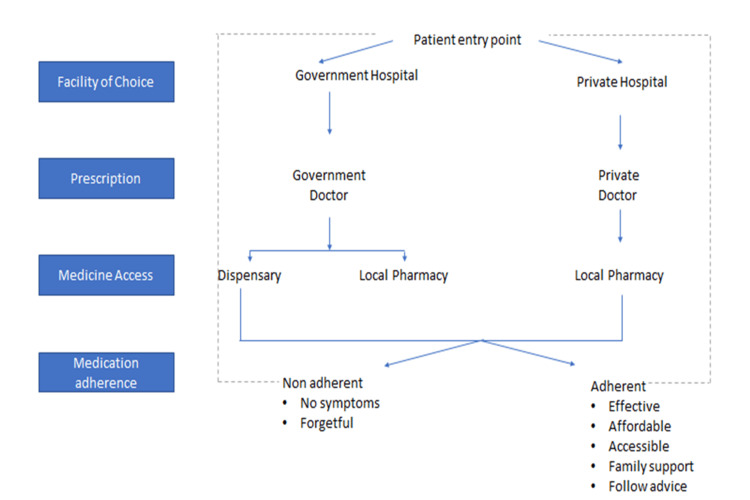
Overview of the patient treatment pathways. Figure credits: All authors.

Most patients reported a common treatment pathway from the government primary health facilities (DGD or Aam Aadmi Mohalla Clinic [AAMC]) due to optimal healthcare accessibility as the prescribed drugs for DM/HTN control were available free of cost at these health facilities (Figure 1). Private pharmacies were utilized by a few patients on rare occasions when the prescribed drugs were unavailable at government healthcare facilities. Treatment preference from private practitioners was observed in very few participants due to shorter waiting times and perception of better efficacy of the prescription in controlling DM/HTN: “At private, the work (physician consultation) is done with less effort and quickly” (P12, F).

Patients' knowledge of DM and self-care

Overall, most of the patients had some awareness of DM. Diabetes was perceived as a disease causing weakness or/and body aches with symptoms such as excessive thirst and hunger. “My legs start feeling weak and my body aches a lot” (P12, F). Another female mentioned, “I stay hungry for food. Also, I encounter dizziness quite often” (P37, F). However, most of the patients lacked an understanding of the etiology of DM in terms of absolute or relative insulin deficiency. “Sugar (DM) is caused by eating sweets…always feeling kind of weak and tired…. sugar can cause complications such as all kinds of ailments like fever, weakness, headache….” (P11, F). Awareness of DM-related complications in the patients was mixed and ranged from no awareness, non-specific (weakness and sickness) to substantial awareness (end organ damage, delayed wound healing, death). “......there may be loss of eyesight and kidneys (also) get affected” (P4, F). The majority of the patients also felt that DM had a significant negative impact on their life and health, leading to frequent bouts of sickness, weakness, hunger, thirst, body pains, and dizziness. “My health has corroded” (P9, F). Control of DM was considered possible apart from medication through a combination of a healthy diet and exercise although their relative importance varied among the patients. “Go for a walk every day, stop eating sweets or sweetened products” (P27, M). A healthy diet was mostly linked to avoiding sweets, and none of the patients reported the importance of concepts of calorie restriction, food exchange, high-fiber diet, etc. “I stopped consuming sweetened food products. I eat them sometimes only” (P42, F). Nonadherence to a healthy diet was frequently attributed to poor glycemic control. “I might not be taking the correct diet and cannot always follow the restrictions” (P17, M). A few patients did know why they were unable to control DM. “I don’t know” (P5, M).

Patients' knowledge of HTN and self-care

HTN was understood by the patients as a complex symptom characterized by headache, weakness, restlessness, vomiting, nervousness, stress, sweating, and an increase in heartbeat. “All I know is that I have a lot of headaches, vomiting and my hands and legs stop working” (P2, F). The etiology of HTN was attributed to several factors such as stress, overthinking, a salty diet, being emotional, and having a family history of the disease. “Well, if you overthink too much your BP level will increase” (P3, M). “...there are many problems in life that cause tension….I feel very tired and sleepy” (P8, F). However, some patients had misconceptions related to the disease's etiology. “I used to take medicines for cough, that could be the only reason (for developing HTN)” (P33, M). Some patients perceived the disease had a negative impact on health and caused serious complications of the kidney, heart, rupturing of vessels (stroke), etc. “The problem is that BP has damaged my eyes, caused painful feet, and headache - if I think of something a little, if there is any tension in the house, there is a fight, then I have a headache” (P7, F). However, occasionally HTN was not construed as a serious disease. “…I do not know much about it… These diseases cannot do anything to the poor...” (P21, F); “Nothing much” (P1, F). Most patients were aware that apart from medication, salt restriction, and physical activity were useful for controlling HTN and its complications. However, none of the patients could differentiate exercise from physical activity, brisk walking from a stroll, and moderate exercise requirement of a minimum of 150 minutes per week. “By taking medicines, also by eating less oily food; also doing regular exercise. Also, less salty food” (P5, M). “When BP rises one must reduce salt consumption” (P13, M).

Medication adherence in DM and HTN: facilitators and barriers

A total of 33 (73%) patients were adherent and 12 (27%) patients were nonadherent to their prescribed medications. All the patients were on treatment and had trust in the efficacy of the medications in controlling DM and HTN. “Only change is now I am surviving because of the medicines. Without these medicines, I do not know what would happen” (P18, F). “if one has to live then he/she has to take medicines…” (P3, M). Forgetfulness was the most common reason attributed to medication nonadherence in nearly one in three patients (Table 2). Family support through reminders and less common payment for medications helped improved their adherence. A majority of patients were unaware of the names of their prescribed anti-DM and anti-HTN medications, but they could identify medicines by physical (shape/color/size) characteristics and correctly reported the frequency of dosing and timing of the medicines. “Small tablet before breakfast. one tablet in the afternoon before lunch, and one in the evening after snacks” (P3, M). Very few patients reported side effects related to anti-DM and anti-HTN medications that were mostly mild (constipation, flatulence, and restlessness) and did not cause any disruption of medication intake. Majority of patients reported never using any alternative medicine to treat DM or HTN. A few patients reported using ayurvedic medicines (an ancient Indian system of medicine) and homemade remedies such as almonds, bitter gourd juice, and neem leaves. Among alternative medicine users, there was consensus on their lack of side effects, but their perceived efficacy for blood sugar or blood pressure control was contested. “Yes, they are pretty effective (for other conditions). But I could not feel their effect when my blood sugar increased. That is why I switched to allopathy” (P22, M). “When the medicines (allopathy) run out of stock, then I (sometimes) take the (ayurvedic) mixture in the morning... It controls my sugar, doesn’t let it increase” (P18, F). None of the patients reported substance abuse or perceived stress as factors that reduce adherence.

## Discussion

This study conducted among patients with DM and HTN attending a primary health facility in a low-income urban area in Delhi assessed patients' perspectives on adherence to self-care practices and their barriers and facilitators. Nearly one in four patients reported medication nonadherence, which is comparable to that observed in other studies conducted in North Indian hospital settings. Most patients could not read medicine labels in the English language due to illiteracy and had to overcome this barrier by memorizing the shape and color of the pills and the associated frequency of dosing.

However, the continuum of care from government PHC facilities in the area, including the DGD, was good due to the regular availability of free-of-cost medications with the absence of significant crowding or queuing, with only a few patients reporting utilizing private pharmacies. Previous studies in LMICs have observed higher utilization of private pharmacies to meet the medicinal needs of patients with NCDs probably due to the difficulty in the accessibility of drugs from the public healthcare system [21,22]. The use of alternative medicines for the management of DM or HTN in the patients was also low due to their lack of perceived usefulness, a finding that corroborates evidence from a large-scale, cross-sectional survey in India.

However, follow-up blood investigations for monitoring glycated hemoglobin, renal function tests, and lipid profile in the patients were poor due to lack of regular prescription by healthcare providers, unavailability of these tests at the PHC facility (DGD), and lack of affordability to spend out of pocket in private laboratories. Similarly, the rates of self-monitoring of blood glucose were low, partly explained by the additional cost of a glucometer and strips that are not provided by public health facilities.

The aforementioned factors are also known to contribute to the phenomenon of therapeutic inertia due to the failure of intensification of medical therapy despite patients not meeting their recommended health targets, a phenomenon also observed in this study. Healthcare providers with limited resources can frequently attribute poor glycemic control to poor medication adherence and avoid treatment intensification, particularly using insulin. The risk of therapeutic inertia is increased in patients lacking up-to-date blood sugar profiles, especially glycated hemoglobin, and in socioeconomically vulnerable populations.

Evidence-based research suggests that lifestyle changes such as a healthy diet and physical activity improve glycemic and blood pressure, reduce the risk of complications, and enhance the quality of life of patients with DM and HTN [21,22]. In this study, awareness of the harmful effects of DM and HTN leading to end-organ damage was inadequate. Similarly, most patients had poor knowledge and understanding of the concept of healthy diet and exercise, findings that are suggestive of the suboptimal patient-provider interaction and deficient counseling in the health facility, reported in both facility- and community-based studies. 

However, none of the patients reported abstinence from tobacco and alcohol as a requirement for the effective management of diabetes and HTN and for safeguarding their health. On the other hand, similar studies conducted in India and Bangladesh have reported on patients' awareness of the need for cessation of tobacco and alcohol to control NCDs [23,24].

The strengths of the study are that it was conducted in a vulnerable urban population in a primary health facility setting. Study limitations include the lack of representativeness of the study sample and its lack of generalizability to other urban or rural settings wherein the predominant treatment-seeking behavior is from the private sector or secondary or tertiary hospital settings. Nevertheless, this study identifies the major barriers and challenges in achieving optimal adherence and health targets in socioeconomically disadvantaged patients undergoing treatment in PHC facilities within resource-limited settings.

## Conclusions

Diabetes and HTN are increasing threats to global public health and are responsible for premature deaths and loss of disability-adjusted life years (DALYs) and quality-adjusted life years (QALYs). Expanding the role of community health workers or volunteers in the prevention and treatment of NCDs and including information regarding nonpharmacological interventions in health promotion packages might help to improve treatment outcomes, adherence, and patient treatment pathways to care.
